# Development of a Controlled-Dose Aerosol Challenge Method Using a Lung Fluid Quantitative Nebulizer

**DOI:** 10.3390/microorganisms14081793

**Published:** 2026-08-14

**Authors:** Mingjie Shao, Huan Cui, Zhaoliang Chen, Lei Zhang, Cheng Zhang, Zhendong Guo, Peng Han

**Affiliations:** 1 College of Life Sciences, Jilin Agricultural University, 2888 Xincheng Street, Changchun 130118, China; 2 Key Laboratory of Jilin Province for Zoonoses Prevention and Control, State Key Laboratory of Pathogen and Biosecurity, Academy of Military Medical Sciences, 573 Tulip Street, Changchun 130122, China; 3 College of Veterinary Medicine, Hebei Agricultural University, 2596 Lucky South Street, Baoding 071000, China

**Keywords:** viral aerosols, influenza virus, pathogenicity, quantitative nebulizer, lung delivery

## Abstract

Airborne transmission represents a primary route of influenza virus dissemination. In animal infection models, conventional intranasal instillation fails to recapitulate the physiological features of natural respiratory infection. Existing aerosol-based challenge methods—including whole-body inhalation and oronasal exposure—face limitations in precise quantification of the delivered infectious dose. Moreover, oronasal exposure systems require specialized, high-cost instrumentation. This study established an economical and dosing-controlled aerosol challenge method utilizing a lung fluid quantitative nebulizer, which can deliver aerosolized virus directly to the lower respiratory tract via endotracheal intubation. The count median aerodynamic diameter (CMAD) of aerosols generated by the nebulizer was 2.28 μm. We compared pulmonary deposition efficiency and pathogenicity between lung-delivery aerosol inhalation and conventional intranasal instillation of PR8 influenza virus in mice. The pulmonary deposition rate for the aerosol inhalation was 63.1%, with an LD_50_ of 31.1 EID_50_; in contrast, intranasal instillation achieved a pulmonary deposition rate of 20.0% and an LD_50_ of 115.0 EID_50_. Histopathological analysis revealed significantly more severe lesions in lungs of mice challenged via lung-delivery aerosol inhalation relative to those receiving intranasal instillation. Furthermore, using a guinea pig model and a recombinant PR8 virus expressing Gaussia luciferase (PR8-Gluc), we demonstrated that pulmonary aerosol inhalation resulted in both more homogeneous spatial distribution and higher viral loads across lung tissues. Our results demonstrate that animals are more sensitive to lung-delivery inhaled virus than virus delivered by intranasal instillation. This study provides an optimized, economical, and technically straightforward aerosol challenge method with controlled administered dosing for viral infection models.

## 1. Introduction

Influenza virus is a ubiquitous respiratory pathogen within the family Orthomyxoviridae, classified as an enveloped, single-stranded, negative-sense RNA virus [1]. Among its types, influenza A viruses (IAVs) display the greatest antigenic and genetic diversity, possess the broadest zoonotic host range, and present the highest pandemic potential—driven by efficient aerosol-mediated transmission and a pronounced capacity for antigenic drift and shift [2,3,4,5,6]. To date, IAVs have been responsible for multiple global pandemics, resulting in substantial morbidity, mortality, and socioeconomic burden worldwide [7]. Consequently, the establishment of etiologically relevant animal infection models remains indispensable for mechanistic investigation of viral infection, pathogenesis, and transmission.

Intranasal inoculation remains the most widely adopted method for establishing influenza virus infection models in laboratory animals, owing to its operational simplicity, precise dosing control, low inter-animal variability, and high experimental reproducibility. However, this route lacks physiological fidelity to natural influenza virus transmission, which can occur via inhalation of infectious respiratory droplets or fine aerosols, as well as through mucosal contact with infectious viruses [8]. Intranasal instillation of viruses does not reflect any of these transmission routes [9]. Studies have confirmed that inhalation of viral aerosols represents one of the primary natural transmission routes for influenza and has been consistently associated with enhanced viral infectivity and pathogenicity [10,11,12,13]. Smith et al. demonstrated that aerosol inoculation with an H3N2 influenza virus in mice caused more robust infection, which was characterized by enhanced morbidity, mortality, pulmonary cell infiltration, and inflammation, compared to intranasal inoculated mice [11]. Similarly, Teunis et al. reported that intranasal inoculation leads to about 20 times lower infectivity than when the virus is delivered in an inhalable aerosol [13]. Collectively, these findings underscore the critical need to develop aerosol-based influenza infection models to accurately recapitulate natural pathogenesis and support robust preclinical evaluation.

Currently, aerosol challenge experiments predominantly utilize two exposure models: whole-body inhalation exposure and oronasal inhalation exposure. The whole-body inhalation exposure model is associated with several methodological limitations. First, since the nebulized agent must fill the entire exposure chamber, large quantities of challenge material are required [14]. Second, the inhaled dose per animal is estimated indirectly—by multiplying spatially limited monitoring-point aerosol concentrations with species- and weight-adjusted minute ventilation and total exposure duration—yet this approach fails to account for intra-chamber concentration gradients, respiratory pattern variability, and dynamic behavioral modulation of inhalation, thereby compromising quantitative accuracy. Third, inter-individual differences in physiological and behavioral parameters—including locomotor activity, body posture, breathing frequency and depth, as well as microenvironmental factors such as localized turbulence, particle settling velocity, and resuspension dynamics—contribute to substantial between-animal variability in actual deposited dose, reflected in high coefficients of variation [9]. Fourth, gravitational settling of viral aerosols onto fur surfaces introduces a confounding contact transmission route, particularly via grooming-mediated oral ingestion, which obscures the attribution of infection to the intended inhalation pathway and undermines route-specific mechanistic interpretation. In comparison, the oronasal inhalation exposure model partially overcomes the limitations of the whole-body inhalation model—particularly pronounced inter-subject variability in delivered dose and ambiguity regarding the primary infection route. Nevertheless, the biologically effective infectious dose reaching target respiratory tissues in experimental animals remains subject to indirect estimation: specifically, by multiplying virus-laden aerosol concentration measured via bioaerosol sampling with species-appropriate minute ventilation and total exposure duration. This estimation cascade entails multiple analytically dependent steps—including aerosol collection efficiency, viral RNA recovery during nucleic acid extraction, and quantitative PCR sensitivity and calibration accuracy—each introducing potential systematic bias; such biases propagate and compound across the cascade, thereby amplifying overall uncertainty in the final dose assignment. Additionally, physicochemical and aerodynamic parameters such as nebulized solution viscosity, aerosol particle size distribution, concentration, aerosol flow rate, and turbulence intensity also influence respiratory deposition efficiency [15,16,17,18,19,20]. Therefore, it remains difficult to precisely quantify the effective infectious dose actually inhaled by animals. Furthermore, the oronasal inhalation exposure system entails inherent technical limitations, including stringent requirements for equipment precision and calibration, setup complexity, cost, prolonged delivery times for high-dose administration, and persistently low pulmonary delivery efficiencies [21].

Pulmonary aerosol delivery has been extensively employed for the targeted administration of vaccines and therapeutics to the lungs [21,22,23,24,25]. This route confers dual advantages: enhanced systemic bioavailability and improved site-specific therapeutic action. The large alveolar surface area—coupled with the thin blood–air barrier—facilitates rapid absorption of active pharmaceutical ingredients into the systemic circulation, circumventing gastrointestinal metabolism and hepatic first-pass effect and thereby increasing bioavailability [26,27]. Moreover, pulmonary vaccination elicits robust mucosal immunity at the primary site of pathogen entry [28,29,30,31], whereas inhaled therapeutics achieve high local drug concentrations at pulmonary lesions, maximizing therapeutic efficacy while minimizing off-target systemic exposure and associated adverse effects [32,33]. Collectively, these attributes establish pulmonary aerosol delivery as a highly advantageous strategy for the prevention and treatment of respiratory diseases. Motivated by the well-documented advantages of pulmonary aerosol delivery, we hypothesize that targeted aerosol administration of respiratory viruses to the lungs may enable the establishment of a more precisely controlled and reproducible animal infection model—thereby enhancing the preclinical evaluation of antiviral efficacy for lung-targeted vaccines and therapeutics.

In this study, we developed an economical and precisely quantifiable aerosol infection method utilizing a lung liquid quantitative nebulizer and conducted a systematic comparative evaluation against the conventional intranasal instillation method. We first evaluated viral deposition efficiency in the lungs, the median lethal dose (LD_50_), and associated pulmonary pathology following infection with influenza virus PR8 in a mouse model. Subsequently, using a recombinant PR8-Gluc virus and a guinea pig model, we comparatively assessed the spatial homogeneity of viral distribution and replication titers in lung tissue after infection via the two challenge methods. This study describes a robust and well-characterized methodology for viral aerosol infection, featuring precisely controlled administered dosing—representing an engineering-optimized approach of pulmonary aerosol delivery to address critical limitations of conventional oronasal and whole-body aerosol exposure systems.

## 2. Materials and Methods

### 2.1. Ethics Statement

All animal experiments were performed in strict accordance with the guidelines of animal welfare of the World Organization for Animal Health. Experimental protocols involving animals were approved by the Animal Care and Use Committee of the Academy of Military Medical Sciences (approval number: IACUC of AMMS-11-2025-18).

### 2.2. Animals

Six-week-old female BALB/c mice and six-week-old female Hartley strain guinea pigs were used in this study. They were purchased from Merial Vital Laboratory Animal Technology Company (Beijing, China) and were randomly assigned to each experimental group. Animals were maintained under strictly controlled environmental conditions, with the temperature set at 24 ± 2 °C, relative humidity ranging from 40% to 70%, and a 12 h light/dark cycle.

### 2.3. Viruses and Culture

The A/Puerto Rico/8/34 (H1N1) strain (abbreviated as PR8) and a recombinant PR8 virus containing a *Gaussia* luciferase gene at the C-terminal of the *NA* segment of the viral genome [34] (abbreviated as PR8-Gluc) were used in this study. During replication, the PR8-Gluc reporter virus stably expresses Gaussia luciferase, which catalyzes light emission upon reaction with its substrate, coelenterazine. Bioluminescence intensity exhibits a strong positive correlation with viral load [34]. Viruses were propagated in 9-day-old specific pathogen-free chicken eggs (Merial Vital Laboratory Animal Technology Company, Beijing, China). Viral titers were determined by the median embryonic infectious dose (EID_50_) using the Reed–Muench method. They were all stored at −80 °C before use. All experiments with the PR8 and PR8-Gluc virus were performed in biosafety level 2 laboratories.

### 2.4. Lung Fluid Quantitative Nebulizer and Aerodynamic Diameter Measurement

The lung fluid quantitative nebulizer (Model HRH-MAG4), mouse laryngoscope, and Mouse Intubation Platform were procured from Beijing Huironghe Technology Co., Ltd. (Beijing, China). Rapid actuation of the high-pressure pusher enabled the nebulizer to generate a stable aerosol from PR8 suspension. Aerosol particle size distribution—including count-, mass-, and surface-weighted metrics—was characterized using an Aerodynamic Particle Sizer Spectrometer (Model APS-3321, TSI Inc., Shoreview, MN, USA).

### 2.5. Lung-Delivery Aerosol Inhalation Challenge

Animals were anesthetized by intramuscular injection of Zoletil 50 (50 mg/kg) into the hind limb. Following anesthesia, mice and guinea pigs were positioned supine on a Mouse Intubation Platform, and transoral tracheal intubation was performed under visualization with a dedicated mouse laryngoscope. The nebulizer needle was advanced to the mid-tracheal region, and viral aerosol generation was triggered by rapid actuation of the high-pressure pusher during peak inspiration. Inhalation exposure volumes were standardized at 50 μL for mice and 200 μL for guinea pigs. All intubation procedures were performed by fully trained, proficient operators. Animals with unsuccessful intubation or tracheal trauma were immediately humanely euthanized and excluded from all subsequent analyses to eliminate procedural bias.

### 2.6. Intranasal Instillation Challenge

Animals were anesthetized by intramuscular injection of Zoletil 50 (50 mg/kg) into the hind limb. Following anesthesia, mice and guinea pigs were inoculated with 50 μL or 200 μL of viral suspension, respectively, administered as discrete droplets gently instilled into both nares under manual restraint.

### 2.7. PR8 Deposition Efficiency in Lungs of Mice

Groups of 6-week-old female BALB/c mice (*n* = 5 per group) were infected via lung-delivery aerosol inhalation or intranasal instillation with 50 μL of PR8 suspension containing 10^4^ EID_50_. To quantify viral deposition in the lower respiratory tract, mice were humanely euthanized within 15 min post-challenge, and lungs were aseptically harvested for quantitative viral titration. The entire lung was homogenized in cold phosphate-buffered saline (PBS) using a TissueLyser II (QIAGEN GmbH, Hilden, Germany) at 30 Hz for 10 min. Following centrifugation at 5000× *g* for 10 min at 4 °C, supernatants were collected, and viral titers were quantified by endpoint dilution assay (EID_50_). Lung deposition efficiency was calculated as the ratio of measured pulmonary viral load (EID_50_ per whole lung) to the administered viral challenge dose (10^4^ EID_50_).

### 2.8. Comparative Assessment of Pathogenicity Following Lung-Delivery Aerosol Inhalation Versus Intranasal Instillation as Routes of Influenza Virus Infection

Groups of 6-week-old female BALB/c mice (*n* = 5 per group) were infected via lung-delivery aerosol inhalation or intranasal instillation with 10-fold serial dilutions of PR8 virus, corresponding to challenge doses of 10^1^, 10^2^, 10^3^, 10^4^ EID_50_. For each challenge route, a corresponding control group (*n* = 5) received an equivalent volume of sterile PBS via the identical administration procedure. Specifically, the control group for the lung-delivery aerosol arm underwent complete endotracheal intubation followed by PBS aerosolization. Body weight was recorded daily for 14 days post-challenge, and survival was monitored concurrently. Mice exhibiting ≥25% loss of initial body weight were humanely euthanized in accordance with predefined humane endpoints.

The median lethal dose (LD_50_) for inhaled and intranasally inoculated PR8 was calculated using probit analysis based on group-specific mortality rates.

To evaluate viral replication kinetics and histopathological changes in the lungs, groups of 6-week-old female BALB/c mice (*n* = 10 per group) were infected via lung-delivery aerosol inhalation or intranasal instillation with 50 μL of PR8 suspension containing 10^4^ EID_50_, while another three mice that were inoculated with equal volume of PBS served as the control group. At 1 and 3 days post infection (dpi), five mice per group were humanely euthanized, and their lungs were aseptically harvested for viral titration by EID_50_ assay. Body weight and lung weight were recorded for each animal to calculate the lung index (lung weight/body weight × 100). The relative lung index was normalized to the mean lung index of the intranasally inoculated group. At 3 dpi, lung tissue samples (*n* = 5 for each infection group and *n* = 3 for the control group) were fixed in 10% neutral buffered formalin, processed for paraffin embedding, and sectioned for hematoxylin and eosin (H&E) staining to assess histopathological lesions. Semi-quantitative histopathological scoring was performed by an independent investigator blinded to sample identity and experimental group assignments. A 0–4 scoring scale was applied based on the distribution range of inflammatory lesions in the lung lobe section, with the following criteria: score 0, no pathological changes; score 1, isolated perivascular inflammatory infiltrates; score 2, perivascular and interstitial inflammatory infiltrates involving <20% of the lobe section; score 3, perivascular and interstitial inflammatory infiltrates involving 20–50% of the lobe section; score 4, perivascular and interstitial inflammatory infiltrates involving >50% of the lobe section.

### 2.9. Bioluminescence Imaging of the Lungs of Guinea Pigs Infected with the PR8-Gluc Through Lung-Delivery Aerosol Inhalation or Intranasal Instillation

Groups of guinea pigs (*n* = 15 per group) were infected via lung-delivery aerosol inhalation or intranasal instillation with 200 μL of PR8-Gluc virus suspension containing 10^4^ EID_50_. Based on prior evidence that influenza viral replication in guinea pig lungs peaks at 2–3 days post-infection [34], we selected 72 h as the observational endpoint and set multiple intermediate time points to capture the dynamic process from initial deposition to peak replication. At 0, 12, 24, 48 and 72 h post-infection, three guinea pigs per group were humanely euthanized; the trachea and lungs were then excised en bloc, and 1 mL of coelenterazine solution (50 μg/mL) was instilled into the lung lumen via the tracheal opening. Following surgical ligation of the trachea, bioluminescence imaging of the lungs was performed using the NightOWL II LB 983 In Vivo Imaging System (Berthold Technologies GmbH & Co. KG, Bad Wildbad, Germany). Subsequently, lung tissue samples were collected for viral titration. To ensure anatomical consistency and intersubject comparability, approximately 0.2 g of tissue was excised from each pulmonary lobe maintaining standardized sampling coordinates across all animals. Homogenization was performed in cold PBS using a TissueLyser II (QIAGEN GmbH, Hilden, Germany) at 30 Hz for 10 min. Following centrifugation at 5000× *g* for 10 min at 4 °C, supernatants were collected, and viral titers were quantified by endpoint dilution assay (EID_50_).

### 2.10. Statistical Analysis

Quantitative data were analyzed with GraphPad Prism 10 software (San Diego, CA, USA). For direct independent comparisons between two groups (e.g., aerosol inhalation vs. intranasal instillation at each time point), an unpaired Student’s *t*-test was applied; Welch’s *t*-test was used when variances were unequal between groups. All of the assays were run in triplicate and are representative of at least 3 separate experiments. The error bars represent the standard deviation. *p*-values less than 0.05 indicated significant differences.

## 3. Result

### 3.1. Aerodynamic Particle Size of PR8 Aerosols Generated by the Lung Fluid Quantitative Nebulizer

**Figure 1:**
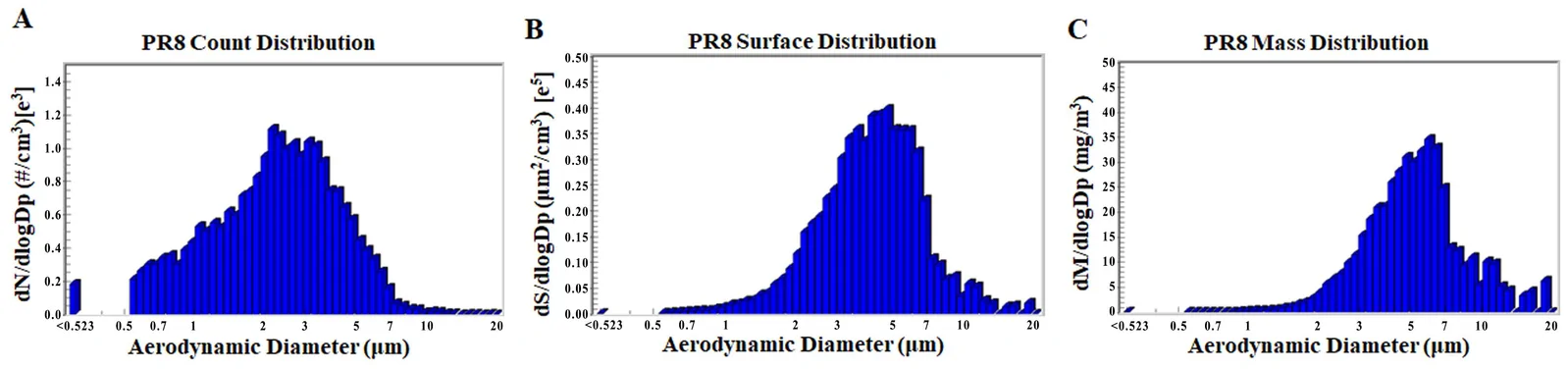
Distribution of particle size count, surface and mass in PR8 aerosols. Aerodynamic Particle Sizer Spectrometer was used to determine the particle size of aerosol generated by the lung fluid quantitative nebulizer. (**A**) The distribution of particle size count; dN is the number of the particles collected in the range (total concentration), and dlogDp is the difference in the log of the channel width. (**B**) The distribution of particle size surface; dS is the surface of the particles collected in the range (total surface) and dlogDp is the difference in the log of the channel width. (**C**) The distribution of particle size mass; dM is the mass of the particles collected in the range (total mass), and dlogDp is the difference in the log of the channel width.

The Aerodynamic Particle Sizer Spectrometer (APS) was used to measure the aerodynamic parameters of PR8 aerosols generated by the lung fluid quantitative nebulizer. Representative plots of the count, surface and mass cumulative particle size distribution are shown in Figure 1. The count, surface and mass median aerodynamic diameter (CMAD, SMAD and MMAD) were 2.28, 4.11 and 5.06 μm, respectively, and their geometric standard deviations (GSDs) were 1.85, 1.64 and 1.62, respectively (Table 1). Further cumulative distribution analysis showed that 60.8% of the total particle count and 7.9% of the total particle mass had aerodynamic diameters below 2.5 μm, while 93.9% of particles by number and 49.5% by mass were smaller than 5.0 μm.

### 3.2. The Lung Deposition Efficiency Was Higher for Lung-Delivery Aerosol Inhalation than Intranasal Instillation

Groups of five 6-week-old female BALB/c mice were infected via lung-delivery aerosol inhalation or intranasal instillation with 50 μL of PR8 suspension containing 10^4^ EID_50_. To quantify viral deposition in the lower respiratory tract, mice were humanely euthanized within 15 min post-challenge, and lungs were aseptically harvested for quantitative viral titration. Results demonstrated that, despite equivalent challenge doses, pulmonary viral titers in the lung-delivery aerosol inhalation group were significantly higher than those in the intranasal inoculation group (Table 2). The lung deposition efficiency of the lung-delivery aerosol inhalation group was 63.1%, approximately threefold higher than that of the intranasal inoculation group (20.0%). These findings indicate that lung-delivery aerosol inhalation significantly enhanced viral deposition in the lower respiratory tract relative to intranasal inoculation.

### 3.3. The Pulmonary Aerosol Delivery Route Confers Greater Pathogenicity to the Virus in Mice Compared with Intranasal Instillation

We compared the pathogenicity of PR8 virus following lung-delivery aerosol inhalation versus intranasal inoculation in a mouse model.

**Figure 2:**
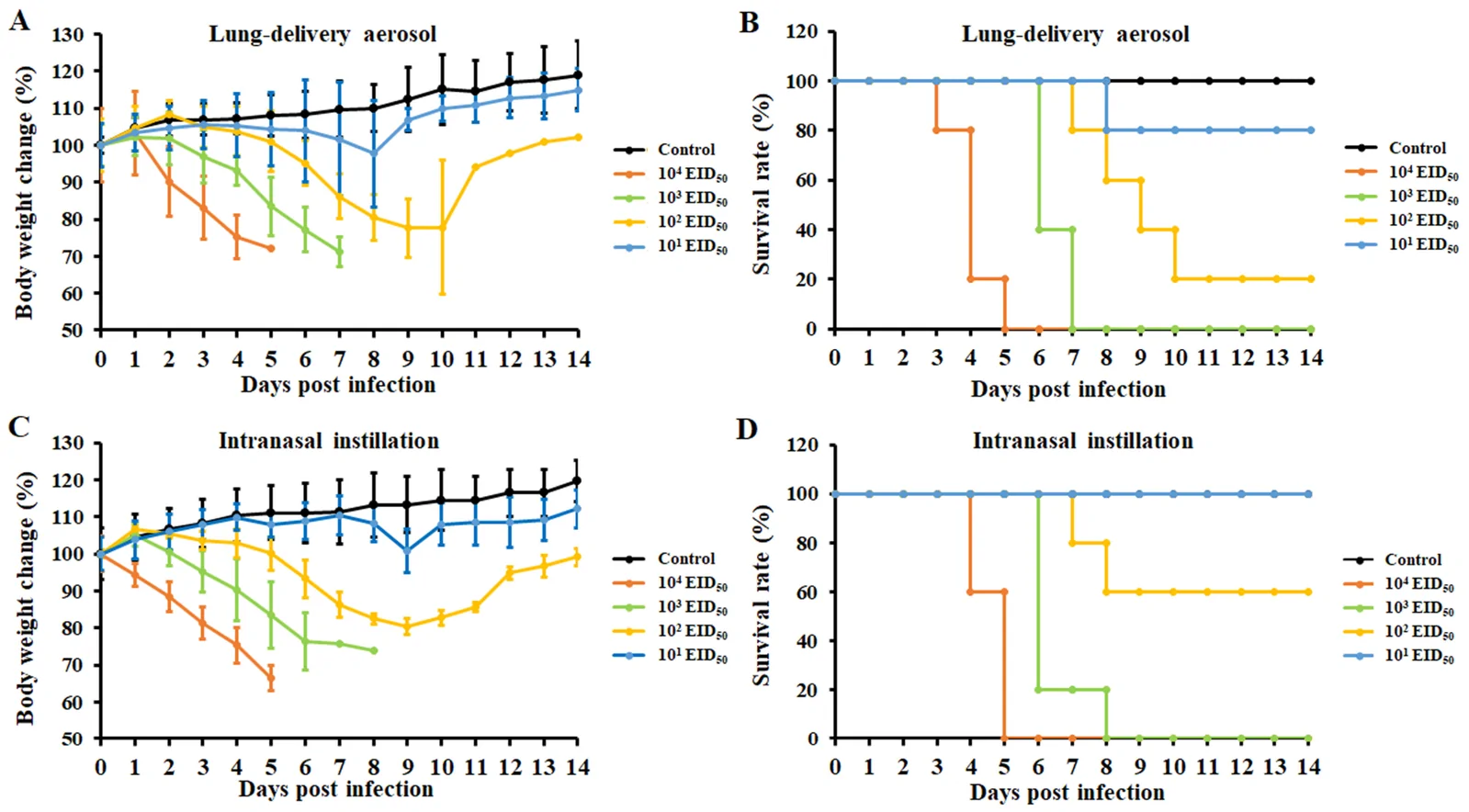
Body weight changes and survival rates of mice infected with the PR8 virus through lung-delivery aerosol inhalation or intranasal instillation. Groups of 6-week-old female BALB/c mice (*n* = 5 per group) were infected via lung-delivery aerosol inhalation (**A**,**B**) or intranasal instillation (**C**,**D**) with 10-fold serial dilutions of PR8 influenza virus, corresponding to challenge doses of 10^1^, 10^2^, 10^3^, and 10^4^ EID_50_. The control group (n = 5) received an equivalent volume of sterile PBS via the same route. Body weight changes (**A**,**C**) and survival rates (**B**,**D**) were monitored daily for 14 days. The mouse body weights were represented as the average weight percentage on the day of infection (day 0). The average body weights of each group were shown and error bars represented the standard deviation (SD).

Groups of five 6-week-old female BALB/c mice were infected via lung-delivery aerosol inhalation or intranasal instillation with 10-fold serial dilutions of PR8 virus, corresponding to challenge doses of 10^1^, 10^2^, 10^3^, 10^4^ EID_50_. The control group received an equivalent volume of sterile PBS via the same route. Body weight changes and survival rates were monitored daily for 14 days. As shown in Figure 2, body weight loss and survival rates following either aerosol delivery or intranasal inoculation of PR8 virus exhibited a clear dose-dependent relationship with the administered viral inoculum. Significant body weight loss was observed for the high-dose infected mice beginning two days post-challenge (Figure 2A,C). In the lung-delivery aerosol inhalation group, lethality rates following challenge with 10^4^, 10^3^, 10^2^ and 10^1^ EID_50_ of PR8 virus were 100%, 100%, 80% and 20%, respectively. The mean time to death for the highest-dose infected mice was 4 days (Figure 2B). In contrast, intranasal inoculation with the same doses yielded lethality rates of 100%, 100%, 40% and 0%, respectively. The mean time to death for the highest-dose infected mice was 4.6 days (Figure 2D).

**Figure 3:**
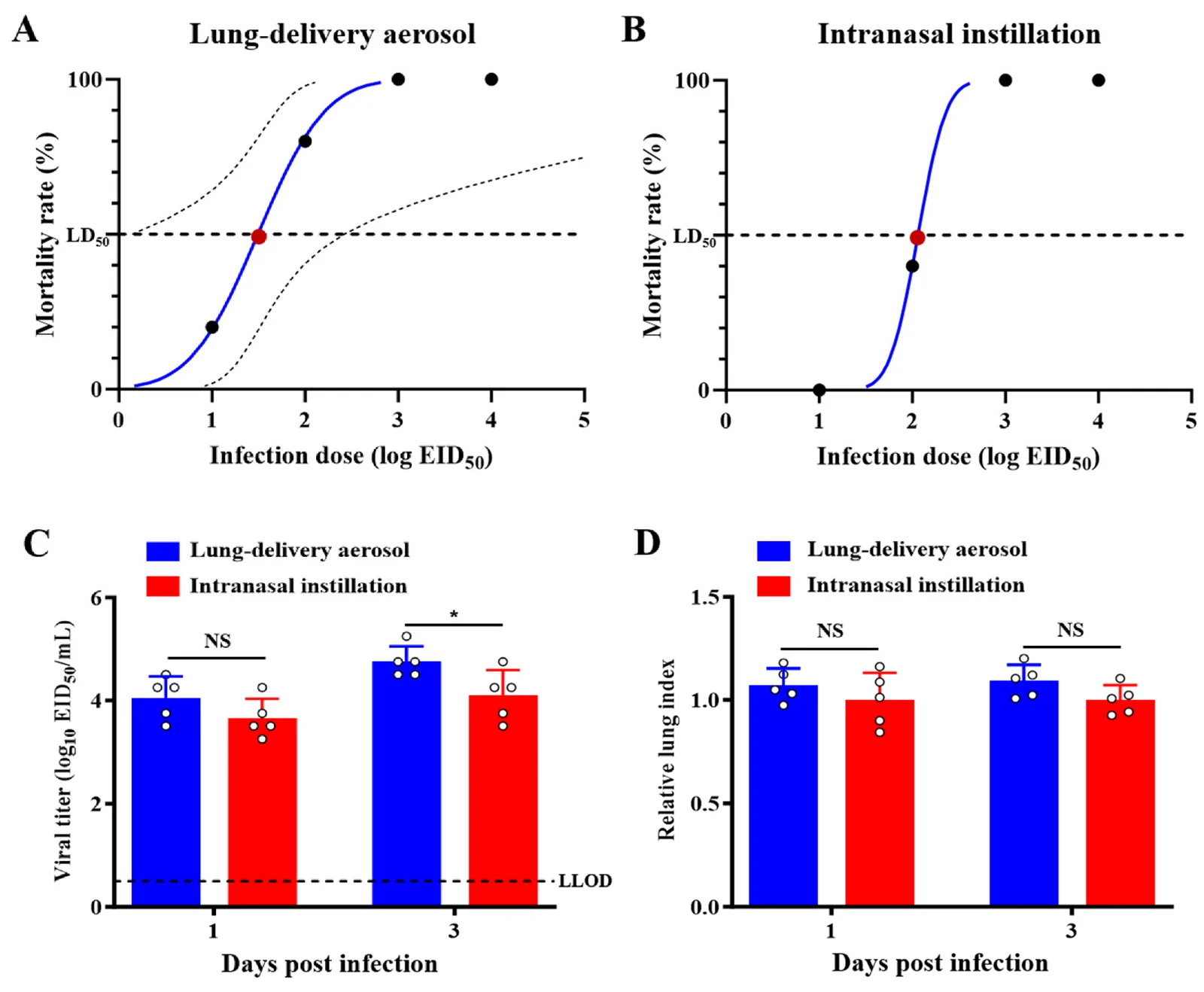
Comparison of LD_50_ and pulmonary viral titers of mice infected with the PR8 virus via lung-delivery aerosol inhalation or intranasal instillation. (**A**,**B**) Probit analysis was used to determine LD_50_ for lung-delivered inhaled (**A**) and instilled (**B**) PR8. Dashed lines show 95% confidence interval for the analysis. A reliable 95% confidence interval could not be calculated for the intranasal instillation group due to limited dose–response data points. (**C**,**D**) Groups of 6-week-old female BALB/c mice (*n* = 10 per group) were infected via lung-delivery aerosol inhalation or intranasal instillation with 10^4^ EID_50_ of PR8 virus. At 1 and 3 days post-infection (dpi), five mice per group were humanely euthanized, and their lungs were collected to determine viral titers (**C**). Body weight and lung weight of each mouse were measured to calculate the lung index. The relative lung index (**D**) was normalized to the mean lung index of the intranasally inoculated group. Data are presented as mean ± SD. Dashed line indicates the lower limit of detection (LLOD). For comparisons of viral titers and relative lung index between the two groups at each time point, an unpaired Student’s *t*-test was applied. NS indicates no significant difference, * *p* < 0.05.

We next determined the LD_50_ for lung-delivery aerosol inhaled, and instilled PR8 by probit analysis. The lung-delivered aerosol-inhaled LD_50_ for mice was estimated to be 31.1 EID_50_, with a 95% confidence interval (CI) of 1.4–263.8 EID_50_ (Figure 3A). The instillation LD_50_ for mice was estimated to be 115.0 EID_50_; however, a reliable 95% CI could not be derived from the current dose–response data, as the limited number of dose groups and sample size prevented full convergence of the probit model (Figure 3B).

We also compared the impact of aerosol-mediated pulmonary delivery versus intranasal inoculation on PR8 influenza virus replication titers and lung index in mouse lung tissue. Groups of ten mice were infected via lung-delivery aerosol inhalation or intranasal instillation with 10^4^ EID_50_ of PR8 virus. At 1 and 3 days post-infection (dpi), five mice per group were humanely euthanized. Body weight and lung weight of each mouse were measured to calculate the lung index. Subsequently, lungs were homogenized in cold PBS for viral titration. Viral titers in lung tissue were significantly higher in mice infected via aerosol-mediated pulmonary delivery than in those infected intranasally, with differences of +0.4 and +0.65 log_10_ EID_50_ at 1 and 3 dpi, respectively (*p* < 0.05 at 3 dpi, Figure 3C). In addition, although no statistically significant difference in lung index was observed between the two groups at 1 or 3 dpi, mean lung indices were numerically elevated in the aerosol-mediated delivery group—by 7% at 1 dpi and 9% at 3 dpi—relative to the intranasal inoculation group (Figure 3D). This trend suggests that inhaled challenges may induce more pronounced pulmonary edema.

**Figure 4:**
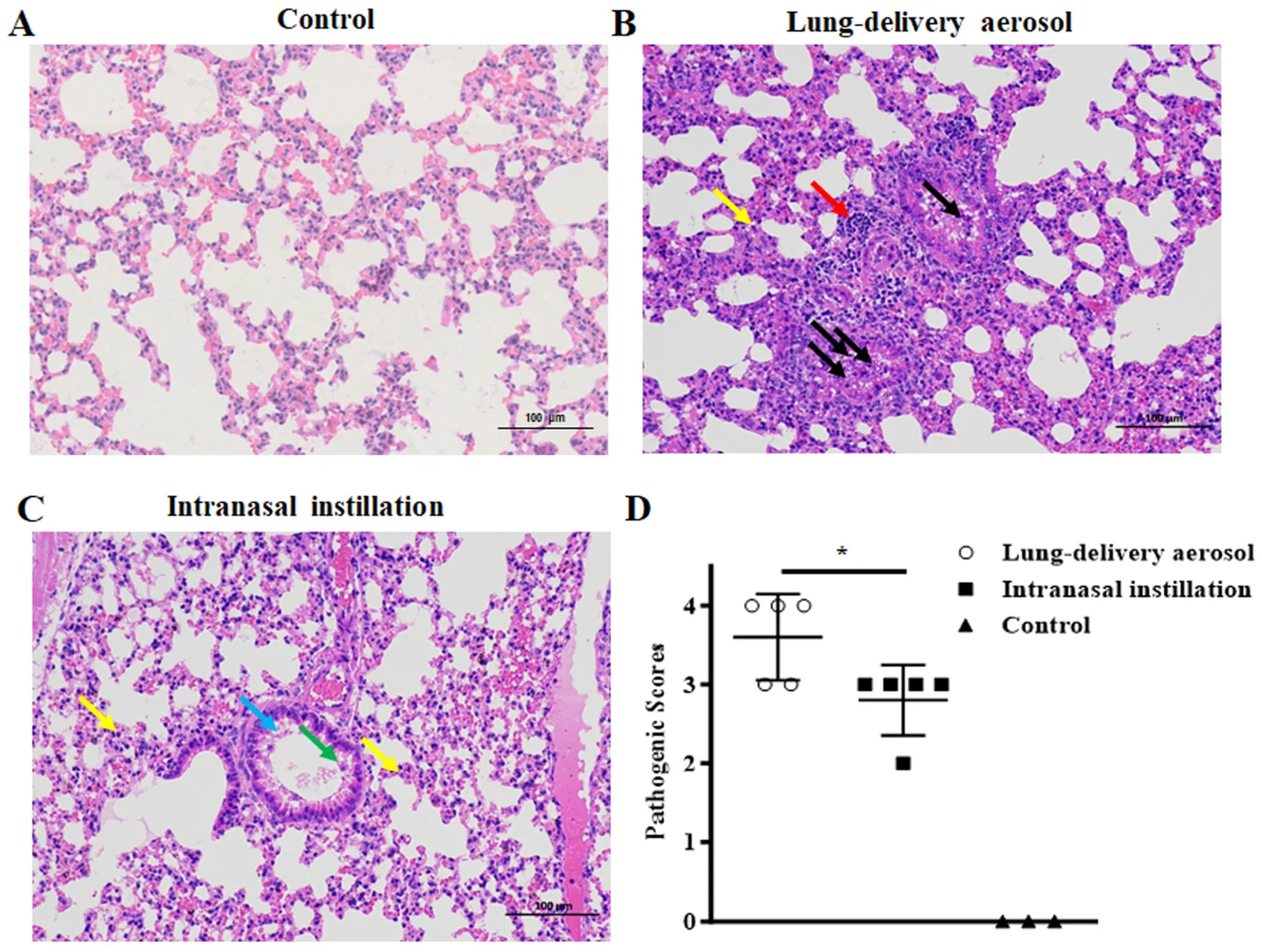
Histopathological analysis of the lungs of mice infected with the PR8 virus through lung-delivery aerosol inhalation or intranasal instillation. Lung histopathology was evaluated at 72 hpi using 5 mice per infection group and 3 mice in the PBS control group. Mice were infected with the PR8 virus through lung-delivery aerosol inhalation (**B**) or intranasal instillation (**C**), and control mice were inoculated with equal volume of PBS (**A**). At 72 hpi, lung tissues were collected from the mice, fixed with formalin, embedded in paraffin, and stained with hematoxylin and eosin. The images were obtained at a magnification of ×20. (Black arrow) Necrotic cellular debris; (yellow arrow) lymphocyte and neutrophil infiltration; (red arrow) perivascular cuffing; (green arrow) epithelial cell detachment; (blue arrow) eosinophilic mucus secretion. The scale bar represents 100 μm. (**D**) Histological scoring for the lungs of PR8-infected mice. Data are presented as mean ± SD. An unpaired Student’s *t*-test was applied to compare the pathogenic scores between the two groups. * *p* < 0.05.

We further examined the histopathological changes of lungs at 3 dpi, and no obvious histopathological changes were observed in the lungs of the control group (Figure 4A). In the lungs of mice subjected to lung-delivery aerosol infection, histopathological examination revealed numerous necrotic cellular debris within bronchial lumens; extensive thickening of alveolar walls accompanied by abundant infiltration of lymphocytes and neutrophils; and discrete perivascular cuffing by inflammatory cells (Figure 4B). In contrast, lungs from mice infected via intranasal inoculation exhibited only focal epithelial cell detachment and minimal eosinophilic mucus secretion in select bronchial lumens and widespread lymphocytic and neutrophilic infiltration along the alveolar walls (Figure 4C). Blinded semi-quantitative scoring further verified the difference in lesion severity between the two groups (Figure 4D). The average histopathological score was 3.60 ± 0.55 in the lung-delivery aerosol inhalation group, which was significantly higher than the score of 2.80 ± 0.45 in the intranasal instillation group (*p* < 0.05). This quantitative result is consistent with the qualitative morphological observations, collectively indicating that lung-delivery aerosol infection induces more severe pulmonary pathological damage.

In summary, the data above indicated that aerosol pulmonary delivery exhibits enhanced pathogenicity of PR8 virus in mouse models relative to intranasal inoculation.

### 3.4. Viruses Delivered via Pulmonary Aerosol Exhibited a More Uniform Spatial Distribution and Higher Viral Titers in Guinea Pig Lung Tissue

**Figure 5:**
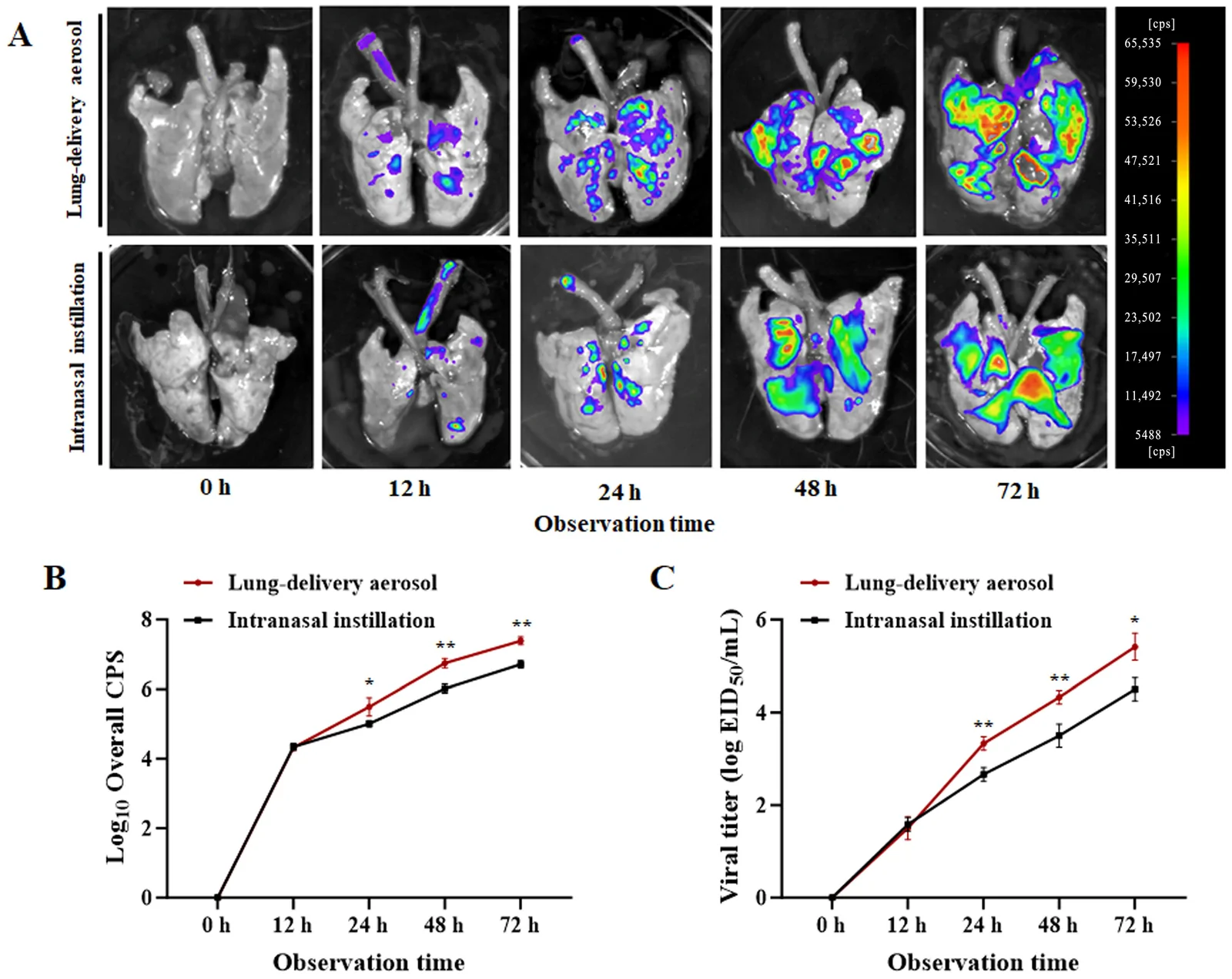
Bioluminescence imaging of the lungs of guinea pigs infected with the IAV-Gluc virus through lung-delivery aerosol inhalation or intranasal instillation. Groups of guinea pigs (*n* = 15 per group) were infected with the IAV-Gluc virus via lung-delivery aerosol inhalation or intranasal instillation. At 0 h, 12 h, 24 h, 48 h, and 72 h post-viral infection, three guinea pigs per group were humanely euthanized, and (**A**) the lungs were dissected and imaged immediately after perfusion of coelenterazine using a NightOWL II LB 983 in vivo imaging system. The color bar on the right shows the intensity of the bioluminescence signal coded in the picture from indigo (5488 counts per second [CPS]) to red (65,535 CPS). (**B**) Overall CPS for the lung bioluminescence imaging results expressed as mean ± SD of three independent experiments. (**C**) Viral titers of lungs expressed as mean ± SD of three independent experiments. For comparisons of bioluminescence intensity and viral titers between the two groups at each time point, an unpaired Student’s *t*-test was applied, * *p* < 0.05, and ** *p* < 0.01.

In order to more intuitively observe the differences between lung-delivery aerosol inhalation and intranasal instillation infection, we developed a lung tissue bioluminescence imaging method using PR8-Gluc virus [34] and guinea pig models. Groups of guinea pigs were infected with 10^4^ EID_50_ of PR8-Gluc virus via lung-delivery aerosol inhalation or intranasal instillation. At 0 h, 12 h, 24 h, 48 h, and 72 h post-viral infection, the lungs were dissected and imaged immediately after perfusion of coelenterazine using a NightOWL II LB 983 in vivo Imaging System. As shown in Figure 5A, PR8-Gluc delivered via pulmonary aerosol exhibited a more uniform spatial distribution in guinea pig lung tissue. We also analyzed the in vivo dynamics of PR8-Gluc infection by bioluminescence signal intensity and EID_50_ assay and found a good correlation between bioluminescence intensity and the viral load measured in the lungs (Figure 5B,C). Whole-organ bioluminescence intensity and viral titers increased progressively from 0 to 72 h post-infection. At 24, 48, and 72 h, both pulmonary bioluminescence intensity and viral titers were significantly higher following aerosol delivery compared with intranasal inoculation. These findings indicate that administration of PR8-Gluc via lung-delivery aerosol inhalation led to more efficient infection of the lower respiratory tract than intranasal inoculation.

## 4. Discussion

In this study, a lung liquid quantitative nebulizer was employed to deliver viral aerosols directly into the lower respiratory tract of experimental animals. Under direct visualization via endotracheal intubation, the nebulizer catheter was positioned approximately midway along the tracheal lumen, and aerosol generation was synchronized with the inspiratory phase of spontaneous respiration to maximize deposition in the pulmonary parenchyma. Aerosol lung-delivery of a virus allows investigators to deliver adequate viral loads directly into the lung without the limitations associated with oronasal inhalation exposure methods [15,16,17,18]. Additionally, aerosol lung-delivery can achieve deep lung delivery without the involvement of other exposure routes and is more reproducible and quantitative than oronasal inhalation exposure in terms of accurate dosing [21]. Moreover, the pulmonary aerosol challenge method offers the advantage of rapid dose administration, particularly when high-dose challenges are required. From an economic perspective, this compact nebulizer-based approach represents a cost-effective alternative to specialized whole-body and oronasal aerosol exposure platforms, which require substantial capital investment in exposure chambers, aerosol generation and monitoring units, and biosafety exhaust filtration systems. Additionally, direct targeted delivery minimizes the consumption of viral inoculum and experimental reagents, further reducing operational costs. However, it should be noted that this endotracheal aerosol delivery method bypasses the upper respiratory tract, including the nasal filtration barrier and nasopharyngeal mucosal immunity. Accordingly, it does not recapitulate the full process of natural respiratory infection, which involves initial upper airway colonization and stepwise progression to the lower respiratory tract. This method is most appropriate for studies focused on pulmonary infection kinetics, lung tissue pathogenesis, and the efficacy of lung-targeted interventions.

The PR8 aerosols generated by the lung fluid quantitative nebulizer exhibited a polydisperse size distribution [35], with CMAD, SMAD and MMAD of 2.28, 4.11 and 5.06 μm, respectively, and corresponding GSDs of 1.85, 1.64 and 1.62 (Table 1). In line with typical log-normal aerosol behavior, fine particles (≤2.5 μm) dominated by number (60.8%) while coarser particles (2.5–5 μm and above) accounted for the majority of total mass (50.5% above 5 μm). Critically, direct aerosol lung-delivery completely bypasses the nasal cavity, which in rodents acts as a highly efficient filter that traps nearly all inhaled particles larger than 3 μm [36]. This bypass allows the full polydisperse aerosol population to enter the lower respiratory tract directly, explaining the high pulmonary deposition efficiency (63.1%) observed in this study (Table 2). Fine particles (≤2.5 μm) primarily deposit in distal bronchioles and alveolar regions via gravitational sedimentation and Brownian diffusion, establishing deep parenchymal infection [34,37]. Meanwhile, coarser particles (>2.5 μm) deposit predominantly in the tracheobronchial tree via inertial impaction, producing widespread infection along the conducting airways. Given the smaller airway caliber of rodents, inertial impaction of coarse particles is pronounced in the lower respiratory tract, leading to broadly distributed infection across all lung regions. This combined deposition pattern is consistent with our observations of homogeneous spatial viral distribution, high pulmonary viral titers, and severe diffuse lung pathology in rodent models.

Pulmonary deposition efficiency is a critical metric for evaluating the performance of aerosol delivery systems. In the murine nose-only inhalation model of influenza virus PR8 infection established by Bowen et al., the measured pulmonary deposition rate was only 0.14% [9]. Likewise, in the guinea pig whole-body exposure infection model developed by Zhang et al., the pulmonary deposition rate reached just 1.34% [34]. Consistent with these preclinical findings, human-focused studies on aerosolized drug delivery—spanning both in vitro cascade impaction and in vivo imaging assessments—report widely variable but generally low pulmonary deposition efficiencies for conventional nebulizers, typically ranging from 1.3% to 30% [16,38,39,40,41,42]. In contrast, the aerosol pulmonary delivery method developed in this study achieves a pulmonary deposition rate of 63.1% (Table 2), representing a substantial improvement over existing approaches. This high-efficiency, dose-controlled platform offers a robust and reproducible methodology for investigating lower respiratory tract infection mechanisms and advancing translational research in pulmonary therapeutics and vaccines.

The influenza virus primarily targets the respiratory tract, with principal sites of infection including the nasal cavity, pharynx, trachea, and lungs. In this study, aerosol-mediated pulmonary delivery markedly enhanced viral deposition in the lungs relative to intranasal inoculation. Consistent with this improved targeting, aerosol administration resulted in significantly higher mortality, elevated pulmonary viral loads, and more severe histopathological lesions compared with intranasal challenge. These findings support the hypothesis that the magnitude of viral deposition in lung tissue is a critical determinant of influenza pathogenicity. This proposition is corroborated by prior evidence demonstrating that influenza virus replicates more efficiently in lungs than in the nasal cavity [37,43]. Kreijtz et al. further demonstrated that intratracheal inoculation of low-pathogenic avian influenza virus induced substantially greater morbidity and mortality in ferrets than alternative routes [44]. Similarly, multiple independent studies have reported increased virulence and infectivity following aerosol exposure compared with intranasal administration [9,11].

We employed a guinea pig model infected with an IAV/PR8 strain engineered to express Gaussia luciferase, coupled with in vivo bioluminescence imaging, to directly visualize viral distribution in the lungs. As shown in Figure 5A, aerosol lung-delivery yielded a significantly more uniform spatial distribution of bioluminescent signal across the lung tissue compared with intranasal inoculation. This enhanced homogeneity suggests deeper and more extensive deposition of the virus throughout the bronchial tree—including distal airways and alveolar regions—thereby facilitating broader cellular tropism and more efficient replication. Consistent with this observation, viral titers in lung homogenates were significantly higher in the aerosol-inoculated group than in the intranasally infected group (Figure 3C and Figure 5C). These findings align with those reported by Zhang et al., who developed an aerosol infection model via whole-body exposure and demonstrated that aerosol inoculation achieves both greater spatial uniformity and higher replicative efficiency in the lungs relative to intranasal administration [34].

This study has three main limitations: First, this aerosol challenge method provides a controlled administered dose and a relatively high pulmonary deposition efficiency, but not a fully validated absolute lung dose. Residual virus in the nebulizer/tubing and viral loads in extrapulmonary tissues such as nose, trachea and stomach were not determined in this study. Second, although the sample sizes in the probit analysis were sufficient to determine LD_50_, they were relatively small. This is reflected by the wide 95% CI of the aerosol group and the inability to compute a stable CI for the intranasal group. Accordingly, the absolute LD_50_ values reported here should be regarded as trend-level estimates rather than definitive quantitative benchmarks. Third, the endotracheal intubation procedure requires operators to undergo systematic training to ensure consistent and atraumatic targeting of the lower respiratory tract. In this study, two dedicated operators performed all intubations following an approximately 2-week training period; technical proficiency was defined as an unsuccessful or traumatic intubation rate ≤ 5% across 20 consecutive mouse procedures. While this approach demands greater technical skill than conventional intranasal instillation, it remains substantially more economical, operationally streamlined, and quantitatively accurate than specialized whole-body or oronasal inhalation exposure systems that require complex instrumentation and indirect dose estimation.

## 5. Conclusions

This study established an economical and dosing-controlled aerosol-based infection method that achieves significantly higher pulmonary deposition efficiency than intranasal inoculation. Consequently, the aerosol method induces enhanced pathogenicity in mice—evidenced by a trend toward a lower LD_50_ value, elevated viral titers in lung tissue, and more pronounced histopathological lung lesions. Moreover, it yields a markedly more uniform spatial distribution of infection throughout the guinea pig lung tissue. Collectively, this well-characterized aerosol infection model constitutes a robust and dose-controlled preclinical platform for the development and evaluation of lung-targeted vaccines and therapeutics.

## Figures and Tables

**Table 1:** Particle size of PR8 aerosols generated by the lung fluid quantitative nebulizer.

Particle Size	Number Particle Size	Surface Particle Size	Mass Particle Size
Media (μm)	2.28	4.11	5.06
Geometric standard deviations	1.85	1.64	1.62
Cumulative % below 2.5 μm	60.8	18.1	7.9
Cumulative % below 5.0 μm	93.9	69.9	49.5

**Table 2:** PR8 deposition efficiency in lungs of mice infected via lung-delivery aerosol inhalation or intranasal instillation.

Infection Method	Virus Concentration (log EID_50_/Mouse)		Percent Deposited ^2^
	Administered	Lung GMT (SD) ^1^	
Lung-delivery aerosol inhalation	4.0	3.8 ± 0.5	63.1%
Intranasal instillation	4.0	3.3 ± 0.4	20.0%

^1^ Titers are presented as arithmetic mean ± standard deviation of log_10_ EID_50_ values from 5 individual mice per group. ^2^ Deposition efficiency is calculated as the ratio of the group mean pulmonary viral titer to the total administered challenge dose (10^4^ EID_50_).

## Data Availability

The original contributions presented in this study are included in the article. Further inquiries can be directed to the corresponding authors.
